# EEG Signal Classification with Data Augmentation for Epileptic Focus Localization and Deep Sleep Detection

**DOI:** 10.3390/s26020474

**Published:** 2026-01-11

**Authors:** Ruixuan Chen, Xin Ma, Xusheng Li, Linfeng Sui, Taiyo Maeda, Qipeng Chen, Jianting Cao

**Affiliations:** 1 Graduate School of Engineering, Saitama Institute of Technology, Fukaya 369-0293, Japan; i3002hgt@sit.ac.jp (R.C.); f4009gma@sit.ac.jp (X.M.); suilf0210@gmail.com (L.S.); maedat@sit.ac.jp (T.M.); 2 Zhejiang XCC Group Co., Ltd., Shaoxing 312500, China; lixushengt@sina.com; 3 College of Information Engineering, China Jiliang University, Hangzhou 310018, China; 4 RIKEN Center for Advanced Intelligence Project (AIP), Tokyo 103-0027, Japan

**Keywords:** epileptic foci, deep sleep, data augmentation, convolutional neural network, k-fold cross-validation

## Abstract

Electroencephalography (EEG) plays a crucial role in clinical neurodiagnostics, particularly in epileptic focus localization and deep sleep detection. However, the limited availability of annotated EEG data hinders the generalization capability of deep learning models. This study proposes a unified EEG classification framework that applies three lightweight data augmentation techniques, namely time shifting, amplitude scaling, and noise addition, to enrich training diversity and enhance model robustness. The framework is evaluated using DeepConvNet, ShallowConvNet, and EEGNet on two public datasets that represent physiological and pathological EEG tasks. Experimental results show that data augmentation consistently improves classification performance across all models and tasks. Importantly, even when baseline accuracies are already high, the proposed augmentation strategies provide additional gains of up to approximately 2.06% in deep sleep detection and 4.07% in epileptic focus localization. These findings demonstrate that simple augmentation methods can effectively improve the robustness and classification performance of EEG-based deep learning models, especially under data-limited conditions.

## 1. Introduction

Electroencephalography (EEG) is a non-invasive technique with high temporal resolution and real-time recording capability [1]. It has been widely applied in neuroscience and clinical diagnostics for analyzing dynamic neural activity. Among its many applications, epileptic focus localization and sleep stage assessment are two clinically important tasks that rely heavily on accurate interpretation of EEG signals [2].

Epilepsy affects more than 50 million people worldwide [3]. While antiepileptic drugs manage symptoms in most cases, nearly one-third of patients suffer from drug-resistant epilepsy (DRE) and require surgical removal of the epileptogenic zone [4]. Accurate identification of this region is crucial for improving surgical success. Although neuroimaging methods such as fMRI, MEG, and intracranial EEG (iEEG) offer valuable diagnostic insights, each has limitations related to invasiveness, cost, or temporal resolution [5]. Scalp EEG remains the most accessible tool for non-invasive seizure monitoring, yet manual annotation of interictal events is time-consuming and vulnerable to subjective variability [6]. These factors highlight the importance of developing automated and reliable EEG-based seizure localization systems [7].

Sleep staging is another essential EEG application, as EEG provides the most accurate representation of sleep dynamics [8]. According to the American Academy of Sleep Medicine (AASM), sleep includes rapid eye movement (REM) and non-REM (NREM) phases, with N3 representing deep sleep characterized by dominant delta activity (0–4 Hz) [9]. Deep sleep plays a vital role in physiological restoration and cognitive processing [10]. However, manual scoring of sleep recordings is labor-intensive and subject to inter-rater inconsistency, especially for long-term monitoring [11]. Thus, automated deep sleep detection is crucial for scalable sleep analysis [12].

Recent advances in deep learning, particularly convolutional neural networks (CNNs), have significantly improved the performance of EEG-based classification tasks [13,14]. CNNs can learn hierarchical spatiotemporal and spectral representations directly from raw EEG data, reducing the reliance on handcrafted features. This ability makes them especially suitable for tasks such as seizure detection and sleep stage classification [15]. However, the performance of CNNs typically depends on large amounts of labeled data for training. Given the challenges of acquiring and annotating EEG recordings, especially in clinical contexts such as iEEG or long-duration sleep studies, limited data remains a key barrier, often resulting in overfitting and reduced generalization [16,17].

A recent survey highlights the growing role of data augmentation in improving performance and robustness across various classification tasks, including EEG analysis [18]. To address the persistent challenges of data scarcity and variability in EEG-based deep learning, data augmentation has emerged as an effective strategy for enhancing model generalization. Recent studies have further explored advanced augmentation approaches such as GAN-based synthetic EEG generation, Mixup strategies, SpecAugment-style time–frequency masking, and physics-driven signal simulation [19,20,21]. These powerful methods typically rely on additional generative models and large training resources. In this work, we focus instead on lightweight and physiologically motivated augmentation techniques—Time Shifting (TS), Amplitude Scaling (AS), and Noise Addition (NA)—which introduce controlled perturbations in the temporal, amplitude, and noise dimensions of EEG signals. These transformations can be directly applied to raw EEG data, diversify training samples, and improve robustness against inherent variations in EEG recordings, thereby providing a complementary perspective to generative and synthetic-data augmentation approaches.

In addition to sharing the EEG modality, epileptic focus localization and deep sleep detection jointly represent two fundamentally different but complementary categories of clinical EEG analysis. Epileptic focus localization primarily relies on detecting transient, spatially localized pathological discharges, whereas deep sleep detection is dominated by global, long-duration oscillatory patterns such as slow waves and spindles [22]. These tasks therefore differ in temporal scale, spatial distribution, and label structure, covering both event-centric and state-centric EEG classification scenarios [23]. Evaluating data augmentation strategies simultaneously on these two representative applications allows us to examine whether an augmentation method generalizes across heterogeneous EEG characteristics and clinical objectives, rather than being specific to a single task. Consequently, the proposed framework serves as a unified and stress-tested testbed for EEG augmentation, bridging seizure-related and sleep-related analysis within a single methodological perspective.

CNN-based architectures remain widely used for EEG classification because of their ability to extract hierarchical spatiotemporal features, as demonstrated in recent cross-subject BCI studies [24]. Building on this foundation, this study aims to systematically examine the effectiveness of combining CNN models with lightweight data augmentation across two representative EEG tasks: epileptic focus localization and deep sleep detection. We conduct experiments on two publicly available datasets, the Bern–Barcelona iEEG dataset and the Sleep-EDF EEG dataset, and employ three representative CNN models (DeepConvNet, ShallowConvNet, and EEGNet) as baseline architectures. Under a unified evaluation protocol, the results show that physiologically motivated augmentations can consistently improve classification performance across tasks and architectures, providing practical evidence and actionable insights across different models and tasks for EEG deep learning under data-limited conditions.

The remainder of this paper is organized as follows. First, we introduce the EEG datasets and experimental environment used in this study. We then detail the data augmentation techniques and convolutional neural network architectures applied across both tasks. Next, we present the 10-fold cross-validation strategy adopted to ensure robust evaluation. Finally, we report and analyze the classification results of all models under different augmentation conditions, followed by a discussion and conclusion of our findings.

## 2. Materials and Methods

### 2.1. Dataset

In this study, we utilize two publicly available EEG datasets tailored for two distinct clinical applications: epileptic focus localization and deep sleep stage detection.

#### 2.1.1. Bern–Barcelona iEEG Dataset

For the epileptic focus localization task, we use the Bern–Barcelona iEEG dataset curated by Andrzejak et al. [25]. The recordings were collected by the Department of Neurology at the University of Bern and include intracranial EEG (iEEG) segments from five patients with drug-resistant temporal lobe epilepsy. During long-term presurgical monitoring, electrodes were implanted to record local field potentials directly from brain regions of clinical interest.

Compared to scalp EEG, iEEG provides higher spatial resolution and improved signal fidelity, which is particularly useful for epileptic focus localization. The dataset contains 3750 labeled two-channel signal segments acquired from either focal (epileptogenic) or non-focal regions. Here, a “signal pair” denotes two simultaneously recorded iEEG channels provided jointly as a two-channel sample; each sample is labeled as either focal or non-focal [26]. Each segment is 20 s long and sampled at 512 Hz, resulting in 10,240 samples per channel.

Following common practice for iEEG analysis, standard signal conditioning (e.g., normalization/denoising) is applied to facilitate reliable model training and evaluation [27]. In our experiments, each two-channel segment is treated as an indivisible input sample for cross-validation and model training.

#### 2.1.2. Sleep-EDF Dataset

For the classification of sleep stages, particularly deep sleep detection, we use the Expanded Sleep-EDF dataset [28], which contains polysomnographic recordings at night of 197 healthy individuals. The dataset includes EEG, electrooculogram (EOG), electromyogram (EMG), and other physiological signals; however, we restrict our analysis to the EEG recordings.

All recordings were annotated by trained sleep technicians according to the Rechtschaffen and Kales manual [29], with sleep stage labels assigned every 30 s. Our analysis focuses on the SC subset, recorded between 1987 and 1991 during a study on sleep and aging in healthy Caucasian adults. EEG signals were sampled at 100 Hz, and no sedative medications were administered. Each subject contributed approximately two 20-h recordings collected over two consecutive day-night cycles in a home environment [30].

For EEG preprocessing, the recordings were band-pass filtered between 0.1–50 Hz to preserve sleep-related frequency components and suppress slow drifts and high-frequency noise. The analysis was performed using the standard EEG channels provided in the Sleep-EDF database; specifically, the Fpz–Cz and Pz–Oz derivations were used for all subjects to ensure consistency across recordings. The EEG signals were segmented into 30-s epochs according to the original hypnogram annotations; epochs containing missing values or obvious recording failures were excluded. Each epoch was then z-score normalized per channel to reduce inter-subject amplitude variability before model training.

In our experiments, we utilized a total of 16,000 EEG segments extracted from the Expanded Sleep-EDF dataset as a balanced subset for efficient and controlled evaluation. This subset consisted of 8000 samples corresponding to deep sleep (N3 stage) and 8000 non-deep sleep samples, evenly distributed across Wake (2000 samples), REM (2000 samples), N1 (2000 samples), and N2 (2000 samples).

These two datasets provide complementary challenges for EEG-based classification, one focusing on abnormal pathological activity (epilepsy) and the other on physiological sleep dynamics.

### 2.2. Data Augmentation

Recent studies have demonstrated the effectiveness of data augmentation in improving EEG model generalization, including GAN-based strategies designed for identity recognition [31]. Building on these findings, we address the challenges of data scarcity and class imbalance in clinical EEG applications—such as epileptic focus localization and sleep stage classification—by employing a set of physiologically plausible augmentation techniques that diversify training samples while preserving the essential temporal and spectral characteristics of EEG signals [32].

Since EEG signals originate from biophysical neural activity and are further shaped by the measurement chain, variability naturally arises from factors such as sensor displacement and timing jitter, electrode–skin impedance fluctuations, and residual environmental/physiological disturbances. In line with the hybrid physics–data-driven view that physically grounded modeling can complement learning-based methods by improving robustness and interpretability [33], we incorporate lightweight perturbations that approximate these dominant sources of physical variability directly in the signal domain.

This study incorporates three commonly adopted yet computationally efficient augmentation techniques: Time Shifting, Amplitude Scaling, and Noise Addition. Specifically, time shifting simulates small temporal misalignment and phase drift induced by synchronization jitter or subtle sensor displacement; amplitude scaling reflects gain/impedance and conductivity-related amplitude variability across subjects/sessions; and noise addition emulates mild stochastic disturbances encountered in practical recordings. These physically motivated perturbations are intended to increase training diversity and encourage CNNs to be less sensitive to minor temporal, amplitude, and stochastic variations [15].

#### 2.2.1. Time Shifting

Time shifting introduces artificial delays by displacing the signal along the temporal axis [34,35]. Let *x*(*t*) denote the original EEG segment and Δt be a randomly selected temporal offset. The augmented signal is defined as:

(1) $$x^{\prime }\left(t\right)=x\left(\left(t+\Delta t\right)\;\mathrm{mod}\;T\right),$$

where *T* denotes the signal length. This operation simulates physiological phase lags and timing jitter caused by sensor displacement, subtle subject motion, or variability in neural oscillation timing.

The choice of small temporal offsets (typically within ±30% of the window length) follows previous EEG augmentation studies and reflects realistic timing variations that occur during acquisition [36,37]. Larger shifts tend to alter the morphology of transient events such as K-complexes, sleep spindles, or interictal spikes, whereas overly small shifts provide insufficient temporal diversity. Thus, constraining Δt to a physiologically relevant range enables temporal perturbation without disrupting the underlying waveform structure. Accordingly, we adopt this commonly used, physiologically plausible offset range as a conservative default, while a more formal sensitivity analysis of Δt is left for future work.

#### 2.2.2. Amplitude Scaling

Amplitude scaling simulates inter-subject variability and changes in electrode impedance by applying a multiplicative factor to the EEG signal [14]. Given a random scaling coefficient α∼U(0.8,1.2), the transformed signal is:

(2) $$x^{\prime }\left(t\right)=\alpha \cdot x\left(t\right)$$

The choice of the interval U(0.8,1.2) follows prior EEG augmentation studies, where small-magnitude amplitude perturbations (typically within ±20%) are commonly used to reflect electrode impedance, conductivity differences, and inter-session variability without substantially altering the underlying oscillatory patterns [36,37].

While overly large scaling factors may change the effective dynamic range and distort band-specific characteristics, too small perturbations may provide limited variability. Therefore, we adopt U(0.8,1.2) as a conservative and literature-consistent setting in this work, and more formal parameter sensitivity/optimization will be explored in future studies.

#### 2.2.3. Noise Addition

To improve resilience against acquisition artifacts and background disturbances, Gaussian noise is injected into the EEG signal [13]:

(3) $$x^{\prime }\left(t\right)=x\left(t\right)+\varepsilon \left(t\right),\;\varepsilon \left(t\right)∼N\left(0,\sigma^{2}\right),$$

where σ is a small standard deviation controlling noise magnitude.

The choice of σ corresponding to 1–5% of the signal standard deviation is consistent with established EEG augmentation literature and mirrors realistic sensor noise levels encountered in clinical and ambulatory recordings [37]. The noise level within this range is intended to provide mild stochastic perturbations and may help improve resilience to baseline wander, muscle artifacts, and environmental interference. Conversely, larger noise levels (e.g., >10%) noticeably distort low-frequency power and degrade sleep-related features such as delta activity, whereas smaller values offer minimal variability. The selected interval thus balances physiological plausibility and effective regularization.

Preliminary sensitivity checks using σ ranges of 0.5–3% and 2–8% confirmed that the augmentation effect remains stable within this neighborhood. These observations suggest that noise addition functions as a mild stochastic perturbation that improves generalization without requiring fine-grained parameter tuning, further supporting the reliability of the chosen noise scale.

#### 2.2.4. Application Scope

**Figure 1:**
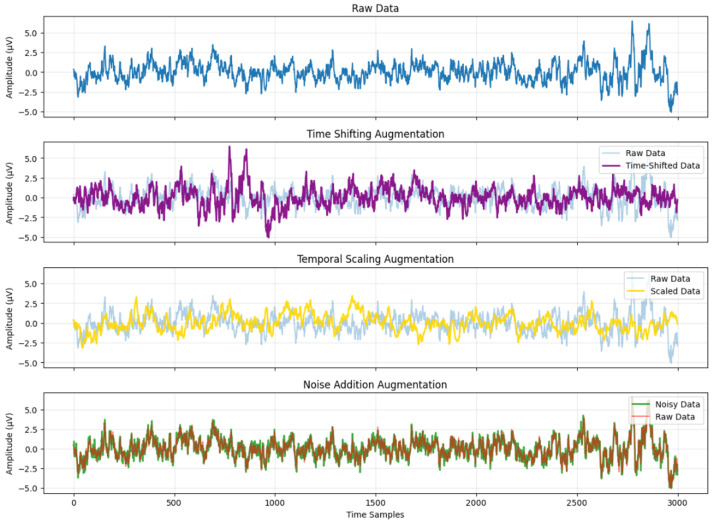
Data after time shifting, amplitude scaling and noise addition.

These augmentation techniques are applied consistently across both the Bern–Barcelona iEEG dataset and the Sleep-EDF EEG dataset. Figure 1 shows representative EEG segments after time shifting, amplitude scaling, and noise addition. In this illustrative example, the time shift is set to 30% of the window length, the amplitude scaling factor is set to 0.8, and Gaussian noise with a magnitude of 5% of the signal standard deviation is added, thereby providing a concrete quantitative example of the augmentation magnitude. The full set of augmentation parameters used in this study is summarized in Table 1. To maximize data diversity and minimize memory footprint, augmentation is performed online during training [38]. From a computational perspective, these augmentations are applied online with simple signal-domain operations (index shifting, scalar rescaling, and additive noise), introducing negligible parameter overhead and only minor runtime overhead compared to CNN forward/backward training. The introduced temporal, amplitude, and stochastic perturbations encourage the CNNs to rely less on exact signal realizations and improve robustness under the adopted evaluation protocol.

In subsequent experiments, we assess the individual and combined impacts of these augmentations across three CNN architectures and two EEG tasks, validating their effectiveness in enhancing classification performance under limited-data conditions.

### 2.3. Convolutional Neural Networks

To effectively model the spatial–temporal characteristics of EEG and iEEG signals in the two target tasks—epileptic focus localization and deep sleep detection—we employ three widely used and representative convolutional neural network architectures: DeepConvNet, ShallowConvNet, and EEGNet [13]. These architectures are selected based on their complementary design philosophies and their proven effectiveness across diverse EEG applications [14].

The rationale for selecting these three models is threefold. First, they span a broad spectrum of architectural complexity: DeepConvNet represents deep hierarchical feature extraction, ShallowConvNet serves as an efficient lightweight baseline, and EEGNet incorporates EEG-specific inductive biases through spatial–spectral filtering. Second, all three architectures have been widely validated in tasks involving seizure analysis, motor imagery, and sleep staging, ensuring that improvements observed from data augmentation are not model-specific but generalizable. Third, their differences in depth, parameter count, and feature focus allow us to systematically examine how augmentation strategies interact with network capacity and inductive biases.

#### 2.3.1. DeepConvNet

DeepConvNet is a deep 1D convolutional architecture designed to capture complex temporal dependencies in EEG and iEEG signals [14]. The network includes five convolutional stages with progressively increasing channel dimensions (32, 64, 128), each followed by batch normalization, ReLU activation, and max-pooling (kernel size 4), enabling multi-level abstraction of high-frequency seizure-related patterns such as spikes and sharp waves.

**Figure 2:**
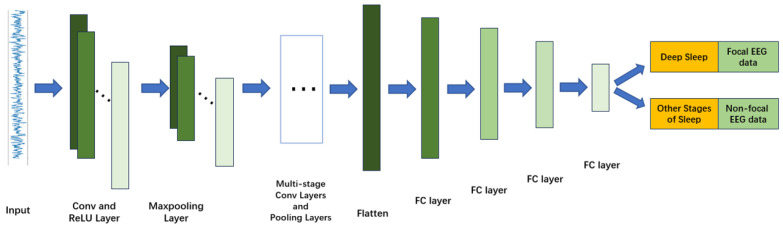
Structure of the DeepConvNet model.

The classifier module incorporates four fully connected layers (5632 → 1024 → 512 → 256 → 128), each paired with batch normalization and dropout, providing strong discriminative capability for subtle inter-class variations. The final linear layer maps the learned representation to task-specific output classes. The architecture is illustrated in Figure 2.

#### 2.3.2. ShallowConvNet

ShallowConvNet adopts a compact, low-complexity design tailored for efficient extraction of fundamental temporal and spatial features. It contains two primary convolutional layers: a spatial convolution that captures channel-level interactions, and a depthwise temporal convolution. These layers are followed by a large-window max-pooling layer (kernel size 35, stride 7), designed to emphasize slow-wave dynamics characteristic of N3 deep sleep.

**Figure 3:**
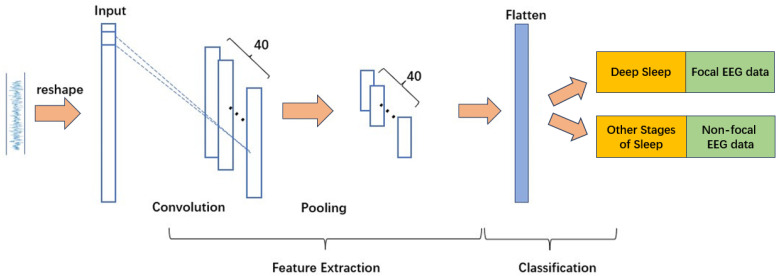
Structure of the ShallowConvNet model.

Its final classification layer is a lightweight fully connected module whose input size is automatically determined based on the input segment length. Due to its low parameter count and simple structure, ShallowConvNet is suitable for real-time applications and resource-constrained deployments. The architecture is shown in Figure 3.

#### 2.3.3. EEGNet

EEGNet is a compact 2D CNN architecture specifically designed for EEG signals, leveraging spatial, spectral, and temporal dependencies through depthwise and separable convolutions [13]. The model begins with a temporal convolution kernel (1×50) followed by a depthwise spatial convolution (with grouped filters F1=8, D=2) that acts as channel-wise spatial filtering. A second block applies depthwise separable convolutions (1×16 kernel), enabling efficient spectral decomposition while maintaining parameter efficiency.

**Figure 4:**
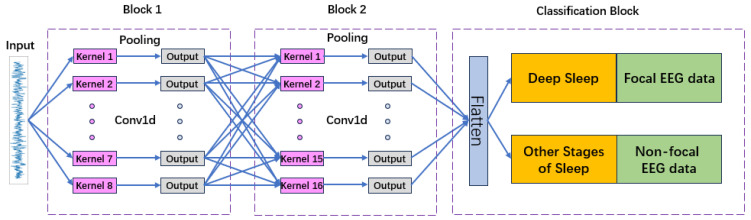
Structure of the EEGNet model.

Dropout layers are incorporated in both blocks to reduce overfitting, and the extracted features are passed into a dense classifier. EEGNet’s strong inductive bias and compact structure enable high performance even with limited training data, making it particularly well suited for both sleep stage classification and seizure detection. The model structure is depicted in Figure 4.

#### 2.3.4. Comparison of Model Architectures

Table 2 summarizes the design differences among the three CNN architectures, including depth, parameter count, and feature emphasis. This comparison highlights their complementary strengths and supports a comprehensive evaluation of model suitability across heterogeneous EEG tasks.

These three CNN models constitute a diverse and well-established benchmark set, enabling a systematic evaluation of how lightweight data augmentation affects model robustness and generalization across clinically relevant EEG tasks.

### 2.4. K-Fold Cross-Validation

In supervised learning, it is important to obtain a reliable estimate of model performance under a specified evaluation protocol. A common pitfall during model training is overfitting, where the model memorizes training patterns but fails to perform consistently on unseen samples (e.g., unseen segments) drawn from the same dataset distribution. To mitigate this issue and to provide a rigorous and stable performance estimate, we adopt the *k*-fold cross-validation technique [39].

Rather than relying on a single train–test split, *k*-fold cross-validation allows each data instance to be used for both training and validation across different folds. This reduces the variance associated with random data partitioning and provides a more reliable estimate of performance [40].

The full dataset D is partitioned into *k* mutually exclusive, approximately equal-sized subsets using stratified sampling to preserve the class distribution:

(4) $$D=\overset{k}{\underset{i=1}{⋃}}D_{i},\;D_{i}\cap D_{j}=\emptyset \;\mathrm{for}\;i\neq j$$

For each fold i=1,2,...,k, we define the training and validation sets as:

(5) $$D_{\mathrm{train}}^{\left(i\right)}=\underset{j\neq i}{⋃}D_{j},\;D_{\mathrm{val}}^{\left(i\right)}=D_{i}$$

The model is trained on $D_{\mathrm{train}}^{\left(i\right)}$ and evaluated on $D_{\mathrm{val}}^{\left(i\right)}$, yielding performance metric $M_{i}$ (e.g., accuracy, precision, recall, or MSE). After *k* iterations, the final cross-validation performance is computed by averaging:

(6) $$M_{\mathrm{CV}}=\frac{1}{k}\sum_{i=1}^{k}M_{i}$$

To quantify the variability induced by different folds and to provide an uncertainty estimate of the reported performance, we report the standard deviation across folds:

(7) $$s=\sqrt{\frac{1}{k-1}\sum_{i=1}^{k}{\left(M_{i}-\bar{M}\right)}^{2}},$$

where $\bar{M}=M_{\mathrm{CV}}$ denotes the mean metric over *k* folds. In addition, we compute the 95% confidence interval (CI) of the mean performance using the Student’s *t* distribution:

(8) $$\bar{M}\pm t_{0.975,\;k-1}\frac{s}{\sqrt{k}}$$

For k=10, $t_{0.975,\;9}=2.262$.

**Figure 5:**
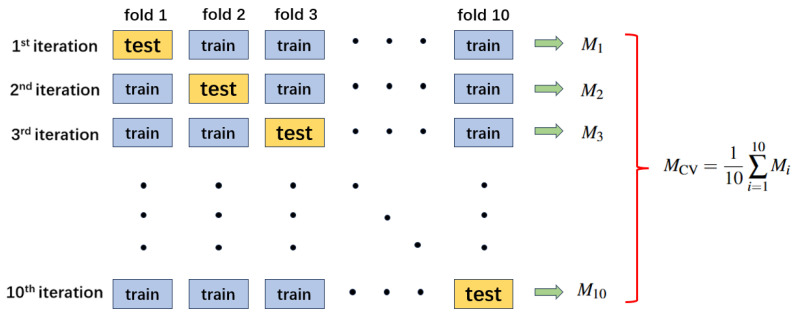
Pipeline of 10-fold cross-validation (K = 10).

In this work, we use 10-fold cross-validation (k=10), a widely used setting that balances bias and variance in evaluation. The data are divided into 10 disjoint subsets. Each subset serves once as the validation set and nine times as part of the training set. Figure 5 illustrates the pipeline of 10-fold cross-validation (k=10).

To ensure fairness and reproducibility:

- The folds are generated using stratified sampling to maintain the class distribution.
- Augmentation is applied online only within each training fold to avoid information leakage into the corresponding validation fold.
- We report accuracy for each fold and summarize the results using the mean, standard deviation, and the derived 95% CI.

This protocol ensures:

- No overlap between training and validation subsets within each fold.
- Performance stability can be assessed across different stratified partitions of the data.
- The mean, standard deviation, and 95% CI provide a quantitative view of split-induced variability under this evaluation setting.

## 3. Results

### 3.1. Device and Experiment

All model training and evaluation procedures were implemented using Python (v3.10) and PyTorch (v2.1.0) on a workstation equipped with a 12-core Intel Core i7 3.50 GHz CPU (Intel, Santa Clara, CA, USA), 128 GB RAM, and an NVIDIA GeForce RTX 2080 Ti GPU (NVIDIA, Santa Clara, CA, USA). This setup enabled efficient handling of high-dimensional EEG data and large-scale experiments.

**Figure 6:**
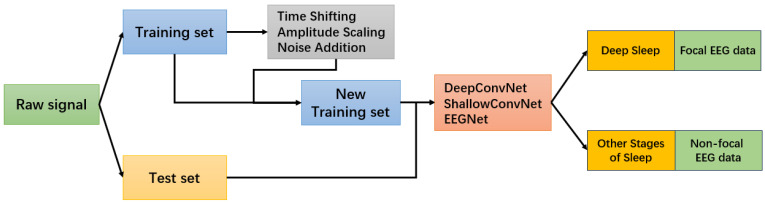
Overview of the training and classification workflow.

Following the preprocessing and augmentation steps described earlier, we trained all CNN models (DeepConvNet, ShallowConvNet, EEGNet) on both datasets using a standardized training pipeline. The complete experimental workflow, including preprocessing, augmentation, model training, and evaluation, is illustrated in Figure 6. We adopted a batch size of 512 and trained for up to 200 epochs on the Sleep-EDF dataset and the Bern–Barcelona iEEG dataset. Early stopping based on validation loss was employed to prevent overfitting.

Data augmentation was applied dynamically during training to enhance model generalization and avoid data leakage. Combined with the 10-fold cross-validation scheme, this ensured robust and fair evaluation across tasks and models. Performance metrics were averaged across folds to report stable and representative results.

To unify the data augmentation, model training, and cross-validation procedures across tasks, we summarize the overall training workflow in Algorithm 1. This unified pipeline ensures consistent evaluation across different CNN models and EEG datasets while incorporating real-time data augmentation.
**Algorithm 1** Unified EEG Training with Augmentation and Cross-Validation**Input:** EEG dataset D, model set $\left\{M_{1},M_{2},M_{3}\right\}$, number of folds k=10, batch size = 512, max epochs *E*
**Output:** Trained models, averaged performance metrics
Split dataset D into *k* folds: $D_{1},D_{2},\ldots ,D_{k}$**For each** model M∈{DeepConvNet, ShallowConvNet, EEGNet}:
–Initialize empty metric list $M_{\mathrm{score}}$–**For** i=1 to *k*:
∗Define training set: $D_{\mathrm{train}}={⋃}_{j\neq i}D_{j}$∗Define validation set: $D_{\mathrm{val}}=D_{i}$∗**For each** signal x(t) in $D_{\mathrm{train}}$:
·Randomly choose augmentation method from {TS, AS, NA}·**If TS:** Apply time shifting using Equation (1)·**If AS:** Apply amplitude scaling using Equation (2)·**If NA:** Add Gaussian noise using Equation (3)·Store augmented signal $x^{\prime }\left(t\right)$ in $D_{\mathrm{train}}^{\mathrm{aug}}$
∗Train model M on $D_{\mathrm{train}}^{\mathrm{aug}}$ using batch size 512 for up to *E* epochs∗Use early stopping based on validation loss∗Evaluate model on $D_{\mathrm{val}}$, obtain metric $M_{i}$∗Append $M_{i}$ to $M_{\mathrm{score}}$–Compute average metric: $\bar{M}=\frac{1}{k}\sum_{i=1}^{k}M_{i}$
**End**


### 3.2. Classification Performance with Data Augmentation

In this section, we report the classification performance of three convolutional neural network models (DeepConvNet, ShallowConvNet, and EEGNet) on two EEG-based tasks: deep sleep detection using the Sleep-EDF dataset and epileptic focus localization using the Bern–Barcelona iEEG dataset. For both datasets, we employed a stratified segment-wise 10-fold cross-validation scheme, following common practice in prior studies and ensuring balanced class distributions across folds. Each model was trained using raw data as well as three data augmentation strategies—time shifting, amplitude scaling, and noise addition—and, in some cases, their combination. All augmentation operations were performed online within the training folds to avoid information leakage into validation sets. The final performance metrics are reported as the mean and standard deviation of accuracy across the 10 folds, and the corresponding 95% confidence intervals (CIs) of the mean can be derived using Equation (8).

Table 3 summarizes the classification accuracies (%) of each model under different augmentation settings. For the Sleep-EDF dataset, DeepConvNet achieved the highest accuracy of 96.07±0.58% with amplitude scaling, while ShallowConvNet and EEGNet reached their best performance under time shifting at 93.69±0.74% and 95.34±0.61%, respectively. The relatively small standard deviations indicate that these improvements are consistent across folds, with time shifting providing the most stable gains among the three architectures. For the Bern–Barcelona iEEG dataset, DeepConvNet reached a peak accuracy of 92.44±0.91% when all augmentation methods were combined, whereas ShallowConvNet and EEGNet achieved 82.93±1.22% and 86.93±1.05%, respectively. Although variances are slightly larger due to the inherent inter-subject variability of clinical iEEG, the results show that augmentation effectively enhances robustness, especially for models with compact feature representations.

For the Bern–Barcelona dataset, applying all three augmentation techniques resulted in a maximum accuracy improvement of 4.05% with the EEGNet model compared to using raw data. Similarly, for the Sleep-EDF dataset, amplitude scaling led to a 2.06% increase in accuracy with the DeepConvNet model. These results suggest that data augmentation—particularly when selecting or combining strategies that match the signal characteristics—can improve classification performance and robustness under the adopted evaluation protocol.

Overall, these results confirm that data augmentation enhances CNN-based EEG classification performance. Among the three methods, time shifting generally provides the most consistent gains, while combining multiple augmentations does not always yield further gains, which is consistent with a regularization–distortion trade-off when perturbations become compounded. These findings suggest that lightweight and physiologically inspired augmentation strategies are effective tools to improve deep learning performance in both pathological and physiological EEG analysis tasks.

### 3.3. Extended Evaluation on Multi-Class Sleep Stage Classification

Deep learning has also been applied to more complex sleep-related tasks, such as CAP phase classification [41], reflecting the increasing interest in automated analysis of sleep microstructures. To further align with this trend and to assess the effectiveness and consistency of the proposed data augmentation strategies, we conducted additional experiments on the Sleep-EDF dataset under different levels of classification granularity. Specifically, we considered 3-class (Wake, NREM, REM), 4-class (Wake, Light Sleep (N1–N2), Deep Slow-Wave Sleep (N3), REM), and 5-class (Wake, N1, N2, N3, REM) settings [42]. The same CNN architectures and augmentation configurations used in the primary experiments were retained to ensure a fair and comparable evaluation across granularities.

Table 4 summarizes the results. Data augmentation consistently improved performance across all settings. In particular, amplitude scaling and the combined augmentation approach yielded notable gains in the 4-class and 5-class scenarios, where class imbalance and inter-stage similarity are more pronounced. These findings demonstrate that the proposed augmentation framework not only enhances binary deep sleep detection but also scales effectively to more fine-grained multi-class sleep stage classification tasks.

In addition to our own experimental results, Table 5 summarizes representative sleep stage classification studies reported in the literature. These works cover a variety of architectures, including SVM-based classifiers, multilayer perceptrons, CNN models, GAN-based generative frameworks, and MixUp-style augmentation strategies, and they span different levels of class granularity from binary to five-class staging. As shown in the table, most existing approaches rely on either complex model designs or task-specific augmentation techniques. In contrast, the present study achieves competitive accuracy using lightweight, physiology-motivated data augmentation applied to standard CNN architectures.

Taken together, these extended experiments verify that the proposed augmentation framework maintains strong generalization ability across various classification complexities and neural network architectures, confirming its applicability to both binary deep sleep detection and more fine-grained multi-stage EEG sleep analysis tasks.

## 4. Discussion

In this study, we systematically investigated lightweight and physiologically motivated data augmentation strategies for EEG classification using CNN-based models across two clinically relevant tasks: epileptic focus localization and deep sleep detection. Beyond demonstrating that augmentation improves model performance, the present results provide additional insight into how and when such augmentation strategies are most effective.

First, the experimental results indicate that different augmentation strategies exert unequal effects on different network architectures. ShallowConvNet and EEGNet benefited most from time shifting and amplitude scaling, suggesting that these models mainly rely on phase and amplitude invariant feature representations. In contrast, the deeper DeepConvNet already achieved strong baseline performance, and augmentation primarily acted as a robustness-enhancing regularizer rather than creating entirely new discriminative structure [32]. These findings imply that the effectiveness of augmentation depends not only on dataset characteristics but also on model capacity and inductive bias, which is consistent with recent observations in EEG deep learning [48].

Second, the observation that combining all augmentation strategies did not always yield the best results indicates the existence of a regularization–distortion trade-off. Moderate perturbations promote invariance learning and reduce overfitting, whereas strong or compounded perturbations may distort physiologically meaningful temporal structures such as sleep spindles, K-complexes, or interictal epileptiform discharges [42,49]. From the perspective of model capacity, compact architectures such as ShallowConvNet and EEGNet may not have sufficient representational flexibility to compensate for multiple heterogeneous perturbations simultaneously, so overly strong augmentation may effectively act as excessive regularization and lead to underfitting. These results suggest that data augmentation should be task-aware and model-aware, rather than treated as universally beneficial in its strongest form.

Third, the augmentation strategies evaluated in this work show consistent benefits on both the Sleep-EDF and Bern–Barcelona datasets, despite differences in clinical objectives, signal characteristics, and recording conditions. This cross-task and cross-dataset consistency suggests that simple, physiology-inspired augmentations can serve as a practical and reliable training enhancement for EEG deep learning under data-limited settings. In practice, this is highly relevant because large, well-balanced EEG datasets are often difficult to obtain in clinical scenarios, and our findings indicate that data efficiency and performance can be improved without modifying model architectures or requiring additional labeled data. These observations motivate considering deployment constraints, where low-cost training enhancements are preferred.

It is also worth noting that the proposed augmentation strategies are computationally lightweight and can be applied directly in the raw signal domain, without requiring generative models or complex training frameworks. This efficiency makes them suitable for real time and resource limited deployments, such as bedside monitoring and mobile EEG systems [17]. Compared with learned or adaptive boosting and augmentation strategies that often require additional optimization stages or multiple learners, our framework achieves performance gains with only minor runtime overhead and without introducing extra trainable components. Importantly, these augmentations are used only during training and do not increase inference complexity. After training, the CNN processes each incoming EEG epoch or window with a single forward pass, which remains compatible with real time operation. In addition, the selected perturbations partially reflect clinically relevant acquisition instabilities, such as mild electrode displacement or contact changes. These effects can manifest as temporal misalignment and gain or impedance fluctuations, and incorporating them during training can improve tolerance to electrode related variability in practical monitoring. Practically, the observed accuracy gains may translate into clinical and monitoring benefits; for deep sleep detection, improved performance can support more reliable home-based and long-term assessment without full polysomnography.

Several limitations should be acknowledged. First, augmentation parameters were selected within physiologically plausible ranges and guided by common practice in prior EEG augmentation studies, rather than being exhaustively optimized through formal sensitivity analysis or robust design procedures [50,51]. Second, the datasets used in this study originate from a limited number of sites, and broader multi center validation remains an important direction for future work. Third, the present study mainly focuses on classification performance and does not explicitly address neurophysiological interpretability. This aspect will be further investigated in future research using model explanation and feature attribution techniques. Moreover, the current setting relies on single channel or limited channel EEG, which restricts spatial interpretability and prevents modeling inter channel topographic patterns. Extending the framework to multichannel EEG will require explicit spatial modeling, for example through learnable spatial filtering, channel attention mechanisms, or graph based modules. Finally, the current evaluation adopts stratified segment wise cross validation and does not strictly enforce subject independent or patient independent partitioning. Therefore, future work will incorporate subject wise and patient wise validation schemes, cross dataset transfer evaluation, multi sensor wearable integration such as combining EEG with MEMS or IoT based human activity recognition, and testing under realistic noise conditions, temporal asynchrony, and distribution shifts. While accuracy is appropriate under our balanced binary setting, future clinically oriented studies—especially for epileptic focus localization—will emphasize sensitivity/specificity (and false-negative rates).

Overall, the results suggest that lightweight, physiologically motivated augmentations can improve CNN-based EEG classification by encouraging representations that are less sensitive to minor temporal, amplitude, and stochastic variations [50]. Beyond methodological interest, these gains may support more reliable clinical monitoring: in deep sleep detection, improved performance benefits home and long-term assessment where wearable EEG often has limited channels and reduced signal quality; in epileptic focus localization, even modest accuracy improvements may help reduce missed or mislocalized foci and thus assist presurgical evaluation [48]. Taken together, the unified evaluation across tasks and architectures provides a practical baseline for enhancing robustness in EEG deep learning, while highlighting when augmentation should be tuned to model capacity and signal fidelity.

## 5. Conclusions

This study shows that three lightweight and physiologically motivated augmentations, namely time shifting, amplitude scaling, and noise addition, consistently improve CNN based EEG classification across three standard architectures and two clinically relevant tasks, including epileptic focus localization and deep sleep detection. The results indicate that these simple signal domain perturbations are particularly effective in data limited settings. They help reduce overfitting and encourage the model to learn features that are tolerant to amplitude variations and temporal phase misalignment, while adding no inference time complexity.

Future work will prioritize validation under subject wise and patient wise splits, as well as broader multi center and cross dataset testing. We will also extend the framework to multichannel EEG by incorporating spatial modeling and interpretable attribution methods, aiming to better support clinician facing deployment under realistic noise conditions and distribution shifts.

## Figures and Tables

**Table 1:** Summary of data augmentation parameters used in this study.

Augmentation	Symbol/Parameter	Range/Setting
Time Shifting	Temporal offset Δt	±30% of window length
Amplitude Scaling	Scaling factor α	α∼U(0.8,1.2)
Noise Addition	Noise standard deviation σ	0.01–0.05×std(x)

**Table 2:** Comparison of CNN Architectures for EEG Signal Classification.

Model	Depth	Parameter Count	Feature Focus
DeepConvNet	Deep (9 conv + 4 FC)	∼1.8 M	Multi-level temporal features
ShallowConvNet	Shallow (2 conv)	∼50 K	Basic temporal–spatial cues
EEGNet	Moderate (2 blocks)	∼100 K	Temporal, spatial, and spectral features

**Table 3:** Accuracy (%) of different CNN models with data augmentation methods on Sleep-EDF and Bern–Barcelona datasets (mean ± std over 10 folds).

Dataset	CNN Models	Raw	Raw+TS	Raw+AS	Raw+NA	Raw+TS+AS+NA
Sleep-EDF	DeepCNN	94.01±0.72	95.46±0.65	96.07±0.58	94.60±0.77	95.60±0.69
	ShallowCNN	92.15±0.88	93.69±0.74	92.39±0.82	92.12±0.81	93.02±0.70
	EEGNet	93.38±0.66	95.34±0.61	95.22±0.63	94.54±0.72	94.87±0.68
Bern–Barcelona	DeepCNN	90.33±1.21	92.12±1.03	90.69±1.15	91.74±1.09	92.44±0.96
	ShallowCNN	79.72±1.48	82.93±1.22	81.20±1.31	81.58±1.29	82.53±1.17
	EEGNet	82.86±1.18	85.96±1.01	83.67±1.22	85.63±1.08	86.93±0.97

**Table 4:** Accuracy (%) of CNN models with data augmentation under 3-class, 4-class, and 5-class settings on the Sleep-EDF dataset.

Setting	CNN Models	Raw Data	Raw+TS	Raw+AS	Raw+NA	Raw+TS+AS+NA
3-Class	DeepCNN	89.12	90.83	91.24	90.67	91.10
	ShallowCNN	87.58	88.91	89.02	88.40	89.31
	EEGNet	88.63	90.14	90.47	89.85	90.70
4-Class	DeepCNN	85.03	86.74	87.65	86.21	87.48
	ShallowCNN	83.69	84.90	85.26	84.60	85.31
	EEGNet	84.25	86.02	86.43	85.80	86.55
5-Class	DeepCNN	81.72	83.64	84.52	82.73	84.25
	ShallowCNN	79.50	80.61	81.00	80.02	81.23
	EEGNet	80.08	82.03	82.76	81.55	82.93

**Table 5:** Summary table of sleep classification models from other relevant studies.

Author	Algorithm	Classes	Accuracy (%)
Nakamura et al. [43]	SVM	Wake, Sleep	95.2
Nakamura et al. [43]	SVM	Wake, NREM, REM	90.0
Hsieh et al. [44]	CNN	Wake, Light sleep, Deep sleep, REM	86.72
Nakamura et al. [43]	SVM	Wake, N1, N2, N3, REM	85.9
Zhang and Guan [45]	MLP	Wake, Light sleep, Deep sleep, REM	85.5
Cheng et al. [19]	GAN	Wake, N1, N2, N3, REM	83.8
Koushik et al. [46]	CNN	Wake, N1, N2, N3, REM	81.72
Aggarwal et al. [47]	MixUp	Wake, N1, N2, N3, REM	81.12

## Data Availability

The EEG datasets used in this study are publicly accessible from the following sources: Bern–Barcelona iEEG Dataset: Available at https://www.upf.edu/web/ntsa/downloads/-/asset_publisher/xvT6E4pczrBw/content/2012-nonrandomness-nonlinear-dependence-and-nonstationarity-of-electroencephalographic-recordings-from-epilepsy-patients (accessed on 26 January 2024). This dataset was used for evaluating the epileptic focus localization task. Sleep-EDF Expanded Dataset: Available at https://physionet.org/content/sleep-edfx/1.0.0/ (accessed on 5 July 2023). This dataset was used for deep sleep stage detection. All additional data generated and analyzed during this study are available from the corresponding author upon reasonable request.
